# Liver fibrosis in biliary atresia: identification of the key gene EDIL3 via integrated bioinformatics

**DOI:** 10.3389/fmed.2025.1726141

**Published:** 2026-01-21

**Authors:** Meng Kong, Jinhua Jia, Chuanyang Liu, Hongzhen Liu, Shisong Zhang

**Affiliations:** 1 Department of Pediatric Surgery, Children’s Hospital Affiliated to Shandong University, Jinan, China; 2 Department of Pediatric Surgery, Jinan Children’s Hospital, Jinan, China

**Keywords:** biliary atresia, bioinformatics, DEGs, EDIL3, liver fibrosis, PI3K-Akt pathway

## Abstract

**Background:**

Biliary atresia (BA) is one of the most destructive liver and biliary diseases in neonates and is characterized by progressive fibrous inflammatory obstruction of the intrahepatic and extrahepatic bile ducts, ultimately leading to liver fibrosis and liver failure. This study aimed to use integrated bioinformatics methods to identify differentially expressed genes (DEGs) in BA liver tissue, identify key genes, and explore their mechanisms in liver fibrosis.

**Methods:**

We obtained data from the gene expression omnibus (GEO) dataset GSE122340 [171 BA patients and 7 normal controls (NCs)]. DEGs were screened via the limma package, followed by gene ontology (GO)/kyoto encyclopedia of genes and genomes (KEGG) enrichment analysis. A protein-protein interaction (PPI) network was constructed via STRING and Cytoscape, and core genes were selected via the maximum clique centrality (MCC), maximum neighborhood component (MNC), and degree algorithms from the CytoHubba plugin. Further focus was placed on the key gene EGF-like repeats and discoidin I-like domains 3 (EDIL3) through gene set enrichment analysis (GSEA), expression validation, subcellular localization analysis, and clinical tissue sample validation. To minimize batch effects, we performed ComBat correction on the combined gene expression data of GSE122340 and the validation dataset GSE46960 before interdataset comparison.

**Results:**

We identified a total of 3706 DEGs, including 2774 upregulated DEGs and 932 downregulated DEGs. The functional enrichment analysis revealed that the DEGs were involved mainly in biological processes such as the cell cycle, DNA replication, and extracellular matrix organization, as well as signaling pathways such as herpes simplex virus infection, phosphoinositide 3-kinase (PI3K)-protein kinase B (AKT), and tumor necrosis factor (TNF). Protein-protein interaction (PPI) network analysis revealed 7 core genes (BRCA1, TOP2A, BRCA2, BUB1B, HSP90AA1, PLK4, and EDIL3), with EDIL3 showing the greatest increase in BA. EDIL3 is located on chromosome 5, and its encoded protein is expressed primarily in the cell membrane and extracellular region. GSEA indicated that high EDIL3 expression was significantly associated with apoptosis and activation of the PI3K-AKT signaling pathway. Clinical sample validation revealed that EDIL3 expression was significantly elevated in BA liver tissue, and its high expression was significantly negatively correlated with the survival rate of patients’ native livers.

**Conclusion:**

Discoidin I-like domain 3 is a novel gene with a potentially key role in BA-related liver fibrosis, possibly influencing the proliferation and apoptosis of cholangiocytes by regulating the PI3K-AKT signaling pathway, thereby participating in the occurrence and development of liver fibrosis. This study provides new insights into the molecular mechanisms and potential treatment strategies for BA.

## 1 Introduction

Biliary atresia (BA) is a devastating neonatal cholestatic disorder, characterized by progressive fibroinflammatory destruction of the bile ducts, which inevitably leads to liver fibrosis and failure, making it the primary cause of pediatric liver transplantation (1, 2). Its global incidence ranges from 1 in 5,000 to 1 in 20,000, and it is most common in Asia (3). Although Kasai portoenterostomy serves as the primary initial surgical intervention to restore bile flow, more than half of patients experience ongoing fibrosis progression postoperatively, necessitating eventual liver transplantation (4). However, even patients who meet the criteria for clearance of jaundice (CoJ) often experience progressive liver injury, leading to various complications such as portal hypertension, cholangitis, hepatopulmonary syndrome, and even malignant tumors (5). The etiology of BA is complex and multifactorial, involving a dynamic interplay of genetic susceptibility, viral infections, immune dysregulation, and abnormalities in biliary development (6–8). Despite extensive research, the precise molecular drivers initiating and perpetuating the fibrotic process remain incompletely understood, significantly hindering the development of early diagnostic tools and effective non-surgical therapies.

In recent years, advancements in high-throughput sequencing technologies and the expansion of public genomic repositories have enabled the application of integrated bioinformatics approaches to systematically dissect the molecular landscape of complex diseases such as BA (9). By analyzing genome-wide expression profiles, researchers can identify differentially expressed genes (DEGs) on a large scale, and subsequently unravel their involvement in key biological processes and signaling pathways through functional enrichment analyses and protein-protein interaction (PPI) network construction (10, 11). Previous molecular studies have highlighted critical roles for immune dysregulation, aberrant cell proliferation, and specific fibrotic pathways in BA pathogenesis. For example, our prior research utilizing weighted gene coexpression network analysis (WGCNA) identified leukocyte cell-derived chemotaxin 2 (LECT2) as a significant gene associated with liver fibrosis in BA, where its elevated expression correlated with poorer native liver survival (12). Other studies have implicated pathways such as the PI3K-AKT and TNF signaling pathways in disease progression (13, 14).

This study aims to conduct an in-depth bioinformatics reanalysis of the BA-related mRNA expression dataset GSE122340 from the GEO database. Notably, we supplemented an independent validation dataset (GSE46960) to confirm the reliability of core gene expression, and integrated clinical follow-up data to clarify the prognostic value of EDIL3, which enhances the clinical translation potential of our findings. Our objective was to systematically identify DEGs in BA liver tissue, pinpoint core regulatory genes within the associated molecular networks, and subsequently focus on a gene demonstrating significant dysregulation yet lacking comprehensive functional characterization in the context of BA. Through this approach, we identified EGF-like repeats and discoidin I-like domain 3 (EDIL3) as the top candidates. EDIL3 was selected for further investigation on the basis of its pronounced upregulation in our analysis, its established roles in extracellular matrix (ECM) biology, cell adhesion, and the regulation of apoptosis; and the PI3K-AKT pathway, which is highly relevant to fibrogenesis coupled with its relatively unexplored status in BA. This study aimed to elucidate the potential role and mechanisms of EDIL3 in BA-related liver fibrosis, thereby providing novel insights into the molecular etiology of BA and potential avenues for future therapeutic strategies.

## 2 Materials and methods

### 2.1 Data acquisition, preprocessing, and screening of DEGs

The dataset GSE122340 was downloaded from the GEO database of the National Center for Biotechnology Information (NCBI).^1^ This dataset is based on the GPL16791 platform and includes mRNA expression profile data from 171 biliary atresia patients and 7 normal controls (15). All the raw data files were processed via the R language. First, the oligo package was used to read the raw CEL files, followed by background correction, quantile normalization, and log2 transformation via the rma method to eliminate technical errors and non-biological variations, ensuring data quality for subsequent analyses. The quality of the processed data was verified via principal component analysis (PCA) and box plots, and no outlier samples were excluded after visual inspection and statistical testing (Grubbs’ test, *P*-value > 0.05). All datasets used in this study are from bulk RNA-seq.

Differential expression analysis was performed via the limma package in R. The processed expression matrix was combined with sample grouping information (BA vs. NC) to construct a linear model. Empirical Bayesian statistics were calculated via the eBayes function. The screening criteria for differentially expressed genes were as follows: adjusted *P*-value (Benjamini–Hochberg method) <0.05 and absolute log2-fold change (|log2FC|) >1. This threshold is widely used in BA transcriptomic studies to balance “biological significance” and “statistical rigor”–a |log2FC| > 1 ensures meaningful expression changes, while adj. *P* < 0.05 was used to control the false discovery rate. Additionally, we performed a sensitivity analysis using a more lenient threshold (|log2FC| > 0.5, adj. *P* < 0.05). The screened DEGs were categorized as upregulated or downregulated genes. A volcano plot was created via the ggplot2 package to display the distribution of all genes, highlighting significantly upregulated and downregulated genes in different colors; a bar chart was also plotted to visualize the number of upregulated and downregulated genes.

### 2.2 GO functional and KEGG pathway enrichment analysis

The list of all screened DEGs was uploaded to the DAVID online database^2^ (v 6.8). GO functional analysis and KEGG pathway enrichment analysis were subsequently performed. GO analysis covered three independent ontologies: biological process (BP), cellular component (CC), and molecular function (MF). A threshold of *P*-value < 0.05 was set. The significantly enriched entries, along with their enrichment scores, gene counts, and *P*-values, were exported from DAVID. In the R environment, the ggplot2 package was used to visualize the top-ranked significant enrichment results, creating facet bar charts and bubble plots, displaying enrichment scores and *P*-values. To avoid redundancy in the enrichment results, we performed hierarchical clustering of the GO terms and KEGG pathways via the clusterProfiler package (version 4.8.3), and retained only the most representative terms/pathways (similarity coefficient > 0.7) for visualization.

### 2.3 Construction of the PPI network and selection of the core genes

The list of DEGs was imported into the STRING database^3^ (v 11.5), setting the organism to “*Homo sapiens*,” with a minimum interaction confidence score > 0.4 and hiding unconnected nodes in the network. The obtained TSV-formatted interaction data were imported into Cytoscape software (v 3.9.1) for network visualization. Cytoscape’s Network Analyzer plugin was used to analyze network topological parameters (node degree, average path length), verify that the network conforms to the characteristics of a scale-free network (R^2^ > 0.8), and ensure the scientific validity of PPI network construction. The CytoHubba plugin in Cytoscape was subsequently used to apply three topological algorithms, namely, maximum clique centrality (MCC), which prioritizes genes in the maximum clique (a subset of fully connected nodes), and is suitable for identifying “core hubs” in dense networks; maximum neighborhood component (MNC), which calculates the maximum number of nodes in the neighborhood of each node, reflecting gene influence on local network structure; and degree, which counts the number of direct interactions of each node, representing basic connectivity. We selected the intersection of the top 10 genes from each algorithm to avoid bias from a single method, ensuring that the core genes were robust.

In the original dataset GSE122340 and another independent validation dataset, GSE46960, the expression levels of the aforementioned core genes in the BA group and normal control group were extracted (11). To reduce batch effects between datasets, we used the sva package (version 3.48.1) to perform ComBat correction on the combined expression matrix of GSE122340 and GSE46960. Statistical analysis of intergroup expression differences for each core gene was performed via R, and their fold changes were calculated. By comparing the expression levels of each gene in the two datasets, the statistical significance of the differences, and the magnitude of the fold changes, along with literature research focusing on genes with relatively few functional studies, the gene with the most significant expression change and greater potential unknown functionality was ultimately selected as the key gene for further in-depth research.

### 2.4 Chromosomal/subcellular localization and GSEA of key genes

The precise chromosomal locations of the key genes were obtained from the NCBI Gene database. Using immunohistochemical and/or mass spectrometry data provided by the Human Protein Atlas database, along with relevant information from the CellMarker database, a comprehensive analysis of the main locations of the encoded protein within the cell was conducted.

The GSEA desktop application (Broad Institute, v 4.3.2) was used. On the basis of the median expression level of the key genes across all samples, the samples were divided into high-expression and low-expression groups. The c2.cp.kegg.v7.2.symbols.gmt gene set was chosen as the reference gene set. The “gene set” permutation type was used for analysis, with 1000 permutations. A nominal *P*-value < 0.05 corresponding to the absolute value of the normalized enrichment score and a false discovery rate (*q*-value) <0.25 were set as the criteria for significant enrichment. The significantly enriched pathways positively correlated with the high expression phenotype of the key genes were the focus of interest. We also performed GSEA via the c5.go.v7.2.symbols.gmt gene set to supplement the functional annotation of EDIL3.

### 2.5 Patient selection and tissue sampling

This study retrospectively included 229 children with BA who underwent Kasai surgery at our hospital from January 2015 to January 2021 as the case group. The inclusion criteria were as follows: age less than 180 days at the time of surgery and liver tissue taken during surgery confirmed by pathological examination as biliary atresia. The exclusion criterion was a lack of complete pathological diagnostic information. Clinical data, including sex, age at surgery, preoperative laboratory test indicators, and pediatric end-stage liver disease scores, were also collected. The control group consisted of 40 patients who underwent surgical resection for hepatoblastoma during the same period, with adjacent tumor tissue confirmed as normal by pathology. The adjacent normal liver tissue was confirmed to have no bile duct obstruction, inflammation, or fibrosis via pathological examination to ensure the reliability of the findings in the control group. All liver tissue samples, approximately 5 mm in size, were taken from the lower edge of the right lobe during surgery, immediately fixed in 4% neutral formaldehyde solution, and then routinely embedded in paraffin. All BA patients were followed up postoperatively, with a follow-up period of 1–60 months. The study endpoint was defined as the status of native liver survival within 5 years post-surgery. The follow-up completeness rate was 92.1% (211/229), and the median follow-up time was 38 months.

### 2.6 RNA extraction and real-time quantitative PCR (RT-qPCR)

Frozen liver tissue samples from 10 BA patients and 10 normal controls were selected. Total RNA was extracted via the TRIzol (Vazyme, China) method, and the RNA concentration and purity were measured. According to the instructions of the reverse transcription kit (Novoproten Scientific, China), 1 μg of total RNA was reverse transcribed into cDNA. Specific primers for EDIL3 and the reference gene GAPDH were designed via Primer-BLAST. The primer sequences for EDIL3 were as follows: forward, 5′-GTTGTGGAGGTTGGTCCCTG-3′ and reverse, 5′-GTGGGCCTGAGCATTTGTAT-3′; the primer sequences for GAPDH were as follows: forward, 5′-GGTGGTCTCCTCTGACTTCAACA-3′ and reverse, 5′-TTTGCTGTAGCCAAATTCGTTGT-3′. Amplification reactions were performed on an RT-qPCR instrument via the SYBR Green (Takara, China) method, with a reaction system of 10 μl. The reaction program was as follows: 95 °C predenaturation for 30 s; 95 °C denaturation for 5 s; and 60 °C annealing/extension for 30 s, for a total of 40 cycles. Each sample was set with 3 replicates. The relative expression level of EDIL3 mRNA in the BA group compared with the control group was calculated via the 2^∧^(−ΔΔCt) method.

### 2.7 Immunohistochemistry (IHC)

Paraffin-embedded liver tissue was sectioned continuously to a thickness of 4 μm. The sections were incubated at 60 °C, dewaxed in xylene, hydrated in gradient ethanol, and then placed in pH 6.0 citrate buffer for antigen retrieval under high pressure for 3 min. After naturally cooling to room temperature, the sample were incubated with 3% hydrogen peroxide solution at room temperature for 10 min to block endogenous peroxidase activity. After washing with phosphate-buffered saline (PBS), normal goat serum was used to block non-specific binding sites for 30 min. The primary antibody [rabbit anti-human EDIL3 polyclonal antibody (Proteintech, China), working concentration of 1:200] was added, and the samples were incubated overnight in a wet box at 4 °C. The next day, after washing with PBS, the corresponding horseradish peroxidase-labeled secondary antibody (diluted 1:1000) was added, and the samples were incubated at 37 °C for 30 min. After washing with PBS, diaminobenzidine was used for color development, and hematoxylin was used to counterstain the cell nuclei, followed by dehydration, transparency, and neutral resin mounting. Negative controls were set by replacing the primary antibody with PBS, and positive controls were confirmed via human placental tissue (known to express EDIL3). Each section was evaluated by randomly selecting 5 non-overlapping fields under a high-power optical microscope. EDIL3-positive signals were located in the cytoplasm and appeared as brown–yellow granules. A semiquantitative scoring system was used: based on the percentage of positive cells (0 points: ≤5%; 1 point: 6%–25%; 2 points: 26%–50%; 3 points: 51%–75%; 4 points: ≥76%) and staining intensity (0 points: no color; 1 point: weak; 2 points: moderate; 3 points: strong), scores were assigned. The two scores were summed to obtain a total score (range 0–7), defined as 0–3 points for low expression and 4–7 points for high expression (12). The consistency between the two pathologists was evaluated via the kappa coefficient (κ = 0.83, *P* < 0.001), which indicated good interobserver agreement.

### 2.8 Statistical analysis

We used GraphPad Prism 9.0 and SPSS 25.0 software for our statistical analysis. Normally distributed data are expressed as the means ± standard deviations, and intergroup comparisons were conducted via independent sample *t*-tests; non-normally distributed data are expressed as medians and were analyzed via non-parametric tests. Count data are expressed as case numbers and percentages, and intergroup comparisons were conducted via chi-square tests or Fisher’s exact tests. We found that EDIL3 levels were closely linked to clinical indicators such as weight, bilirubin, alanine aminotransferase, albumin, lactate dehydrogenase, the international normalized ratio (INR), the neutrophil count, the native liver survival time, and the pediatric end-stage liver disease (PELD) score. Kaplan-Meier survival curves were plotted, and intergroup comparisons were conducted via log-rank tests. Variables selected via univariate analysis were included in a Cox proportional hazards model to identify independent factors, with *P* < 0.05 considered statistically significant. All the statistical tests were two-tailed, and multiple testing corrections were performed where appropriate.

## 3 Results

### 3.1 DEG identification results

**FIGURE 1:**
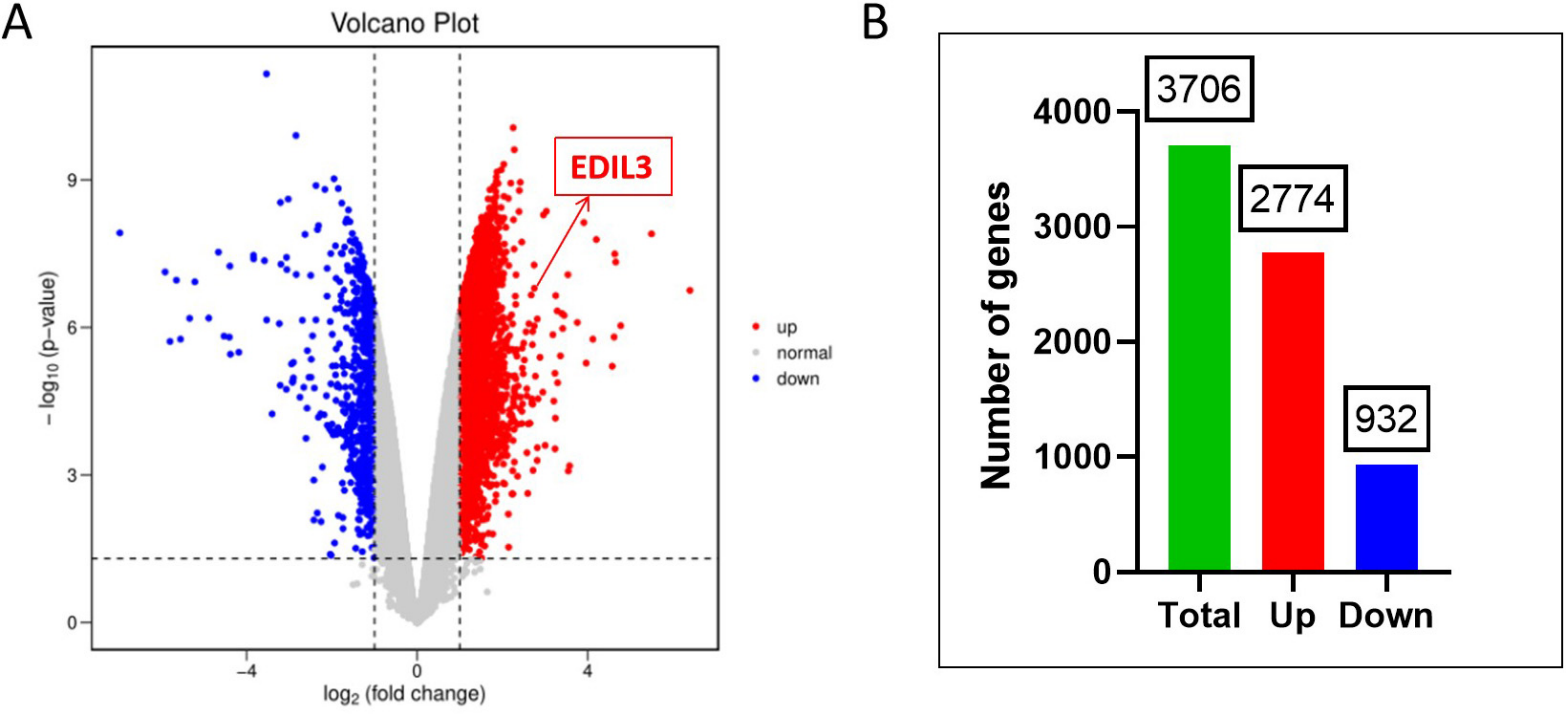
Identification results of DEGs. **(A)** Volcano plot of the differentially expressed genes. The *x*-axis represents the log base 2 of the fold change, and the *y*-axis represents –log10: the negative logarithm of the *P*-value with base 10. The gray points represent genes with no differential expression, the blue points represent downregulated genes, and the red points represent upregulated genes. **(B)** Bar chart of differentially expressed genes.

Through differential expression analysis of the GSE122340 dataset, we identified a total of 3706 genes that were significantly differentially expressed between the biliary atresia group and the normal control group. Among them, 2774 genes were upregulated, and 932 genes were downregulated (Supplementary Table 1). The top 10 upregulated DEGs included EDIL3, PLK4, and TOP2A, whereas the top 10 downregulated DEGs included CYP3A4, CYP2C9, and ABCB1 (Supplementary Table 1). The volcano plot clearly displays the distribution of all genes, with significantly upregulated and downregulated genes clustering on either side (Figure 1A). The bar chart visually shows the comparison of the number of upregulated and downregulated genes (Figure 1B), indicating widespread transcriptomic dysregulation in BA liver tissue, predominantly with upregulation.

### 3.2 Results of the GO and KEGG enrichment analyses

**FIGURE 2:**
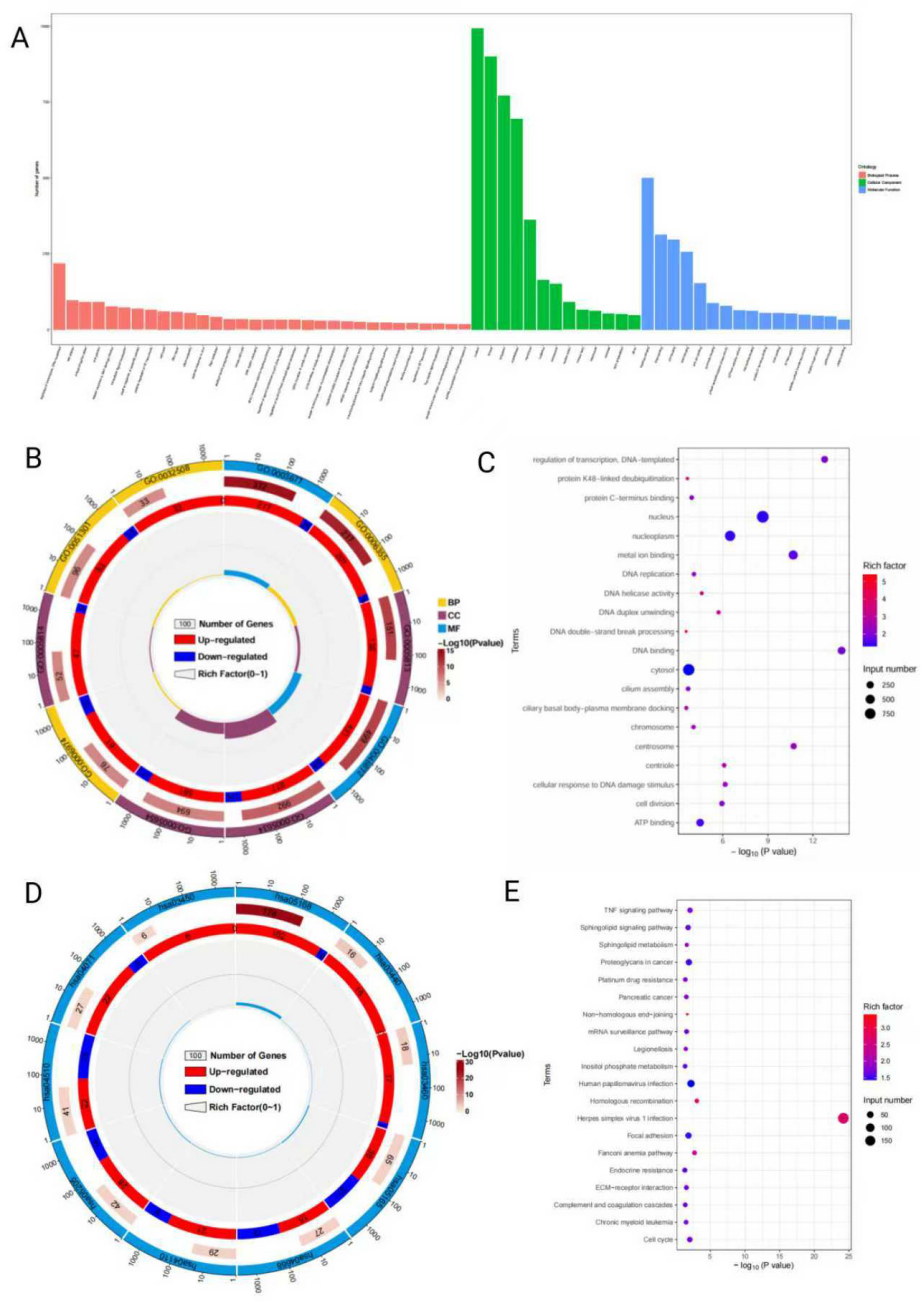
Gene ontology (GO) and KEGG enrichment analyses of DEGs. **(A)** GO enrichment bar chart: the *x*-axis shows GO terms, and the *y*-axis indicates the number of enriched genes. Colors represent categories: BP, MF, or CC. **(B)** GO enrichment circle chart: results are displayed in three concentric circles. The outer ring shows GO term names and categories (BP in yellow, CC in purple, MF in sky blue), with gene counts per term. Color intensity indicates –log10(*P*-value). The middle ring shows gene expression (red: up, blue: down). The inner ring shows the enrichment score (foreground/background gene ratio). **(C)** GO enrichment bubble chart: the *x*-axis shows –log10(*P*-value) (enrichment significance), and the *y*-axis lists GO terms. Dot size corresponds to the number of DEGs; color reflects the rich factor. Top 20 terms by *P*-value are shown. **(D)** KEGG enrichment circle chart. **(E)** KEGG enrichment bar chart: top 20 pathways by *P*-value.

Gene ontology functional enrichment analysis yielded a total of 245 significantly enriched terms (Figures 2A–C). In the biological process category, DEGs were significantly enriched in transcription regulation (DNA template), response to DNA damage stimulus, cell division, DNA replication, and extracellular matrix organization. In the CC category, DEGs were enriched mainly in chromosomal regions, centrosomes, and nucleoplasm structures. In the molecular function category, these genes were significantly enriched in DNA binding, metal ion binding, and ATP binding functions (Supplementary Table 2).

Kyoto encyclopedia of genes and genomes pathway enrichment analysis revealed that the DEGs were significantly enriched in the top 10 pathways (Figures 2D, E), which involved mainly herpes simplex virus infection, human papillomavirus infection, the cell cycle, the TNF signaling pathway, the ECM-receptor interaction, and the PI3K-AKT signaling pathway (Supplementary Table 3). These results suggest that biological processes such as viral infection, the immune inflammatory response, cell cycle regulation abnormalities, cell death, and extracellular matrix remodeling may play key roles in the pathogenesis of BA.

### 3.3 Construction of the PPI network and core gene selection

**FIGURE 3:**
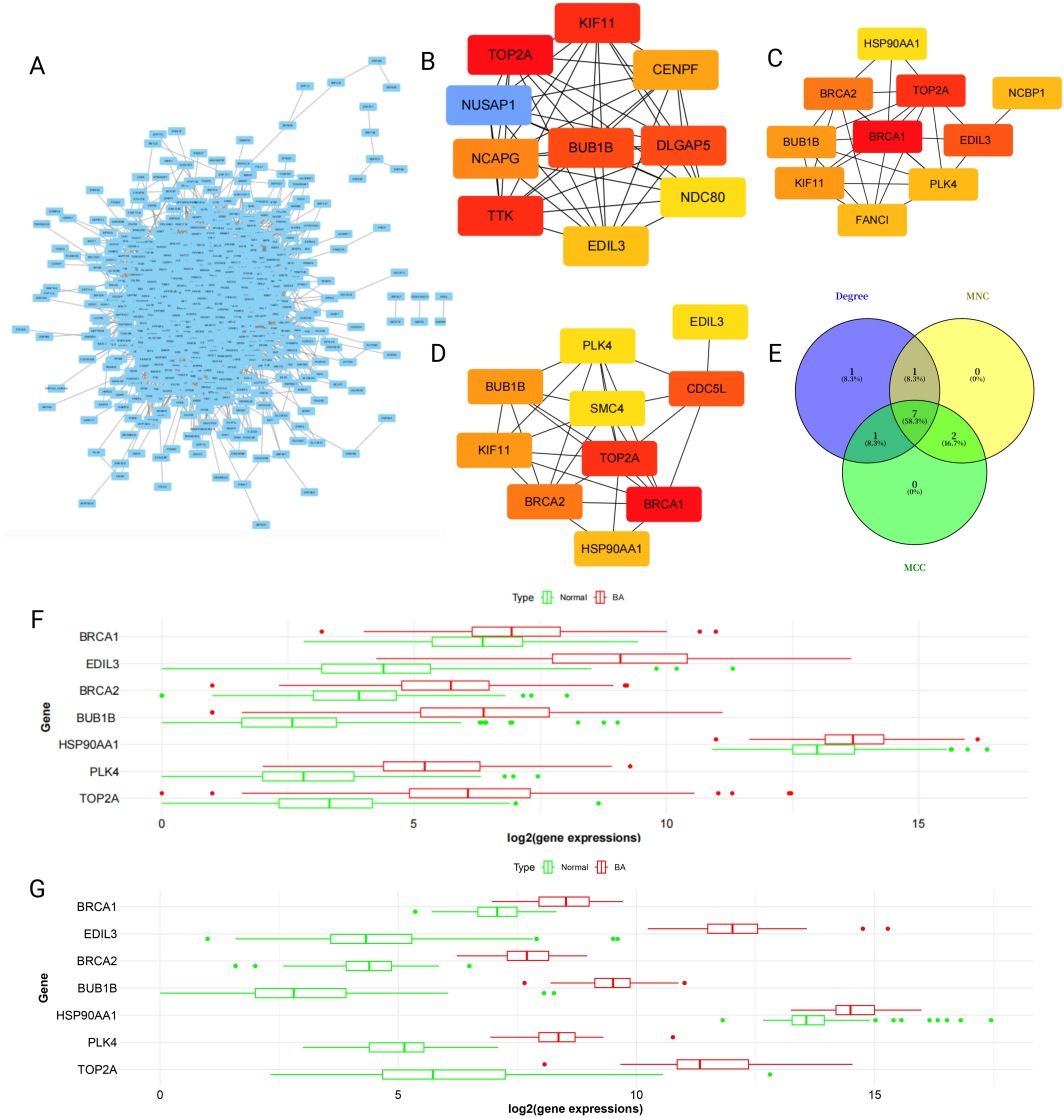
Protein-protein interaction (PPI) network construction and key gene screening. **(A)** PPI network of 3706 DEGs, with nodes representing proteins and edges representing interactions. **(B–D)** We used three algorithms to screen for the top 10 genes with the highest scores: MCC, MNC, and degree. **(E)** Seven core genes were identified. **(F,G)** Key genes tended to be upregulated in both the training set BA group and the validation set, with the EDIL3 gene showing the greatest fold change.

After the 3706 DEGs were imported into the STRING database, a PPI network containing numerous interaction nodes was successfully constructed (Figure 3A). After the network was visualized via Cytoscape, the number of core nodes was calculated via the MCC, MNC, and degree algorithms from the CytoHubba plugin (Figures 3B–D). The top 10 genes from each algorithm were subjected to intersection analysis, ultimately identifying 7 common core genes: BRCA1, TOP2A, BRCA2, BUB1B, HSP90AA1, PLK4, and EDIL3 (Figure 3E). The functions of these genes are related mainly to DNA damage repair, cell cycle checkpoint regulation, chromosomal separation, and protein stability.

In both the original dataset GSE122340 and the independent validation dataset GSE46960, the expression levels of the 7 core genes were validated. Statistical analysis confirmed that all 7 genes had significantly higher expression levels in BA patient liver tissues than in normal control liver tissues. Among them, the difference in the expression of the EDIL3 gene was the most prominent in both datasets (Figures 3F, G). While BRCA1/2 and TOP2A are well-known to be involved in DNA repair and cell cycle regulation (16–18), EDIL3 is a secreted ECM protein with emerging roles in inflammation, angiogenesis, and integrin-mediated signaling processes that are highly relevant to BA pathogenesis. Its significant upregulation, combined with limited prior research in BA and enrichment in fibrosis-related pathways (PI3K-AKT), make it a compelling candidate for further investigation. Furthermore, at present, there are relatively few reports on the role of EDIL3 in BA. Given its significant expression differences and potential research value, we ultimately chose EDIL3 as the key gene for our research target.

### 3.4 Chromosomal/subcellular localization and GSEA results for the EDIL3 gene

**FIGURE 4:**
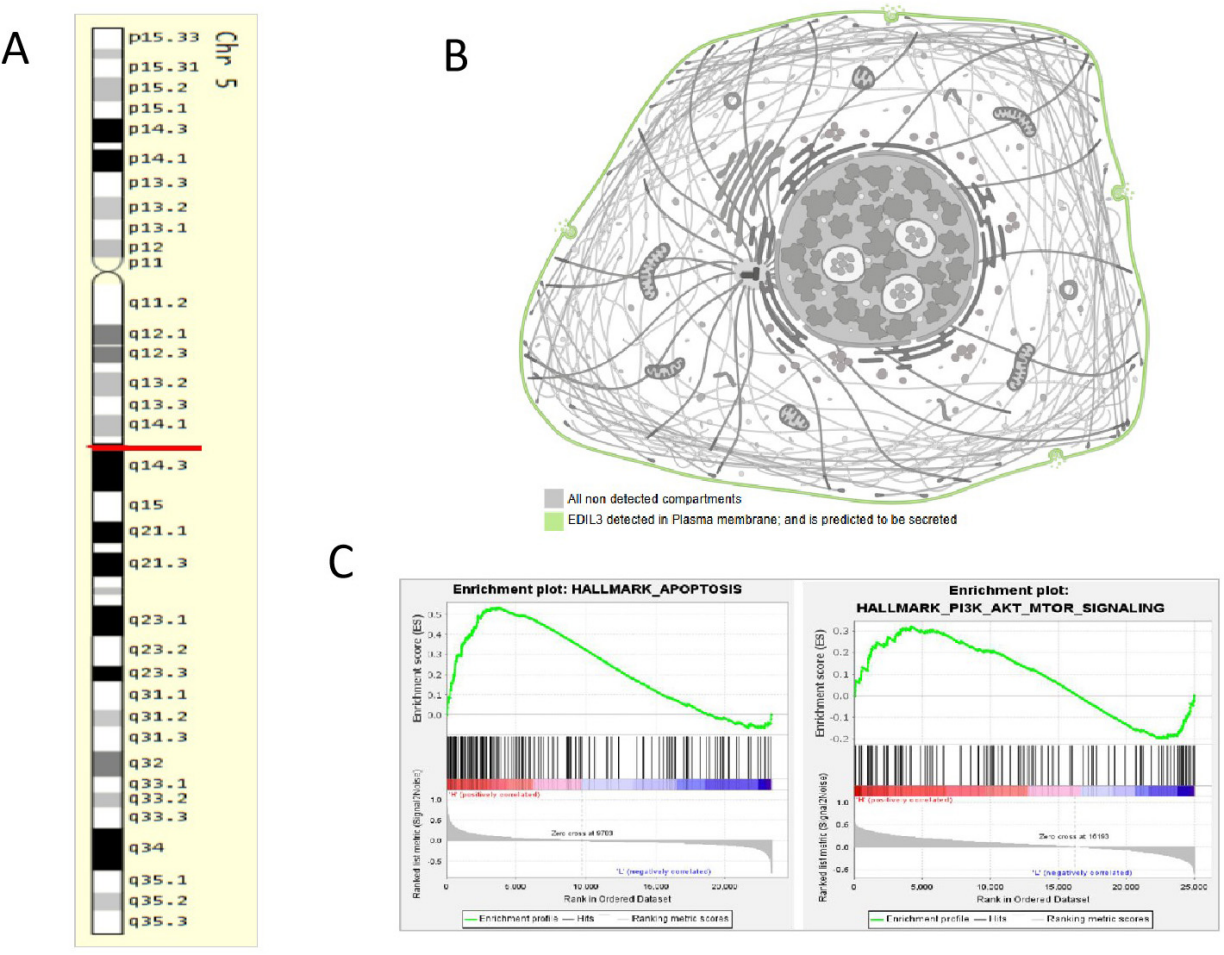
Localization of the EDIL3 gene and GSEA results. **(A)** EDIL3 is located on chromosome 5. **(B)** EDIL3 is found primarily on the cell membrane and is considered a secretory protein. **(C)** GSEA results for EDIL3, indicating that EDIL3 is associated with apoptosis and the PI3K-AKT signaling pathway.

The EDIL3 gene was confirmed to be located on the long arm of human chromosome 5, band 14.1 (5q14.1) (Figure 4A). Subcellular localization analysis, which integrates information from the Human Protein Atlas and other databases, revealed that the EDIL3 protein is a secretory glycoprotein primarily localized to the cell membrane and extracellular region (Figure 4B), which is consistent with its known functions in cell adhesion and signal transduction (13, 14). To explore the biological pathways potentially affected by high EDIL3 expression, GSEA was conducted, revealing that the apoptosis pathway and PI3K-AKT signaling pathway were significantly enriched in BA samples with high EDIL3 expression, and the enrichment scores indicated that these pathways were activated in the high EDIL3 expression group (Figure 4C). These findings suggest that EDIL3 may participate in the determination of the fate of cholangiocytes and the process of liver fibrosis by positively regulating apoptosis and the PI3K-AKT signaling pathway.

### 3.5 High expression of EDIL3 in BA liver tissue

**FIGURE 5:**
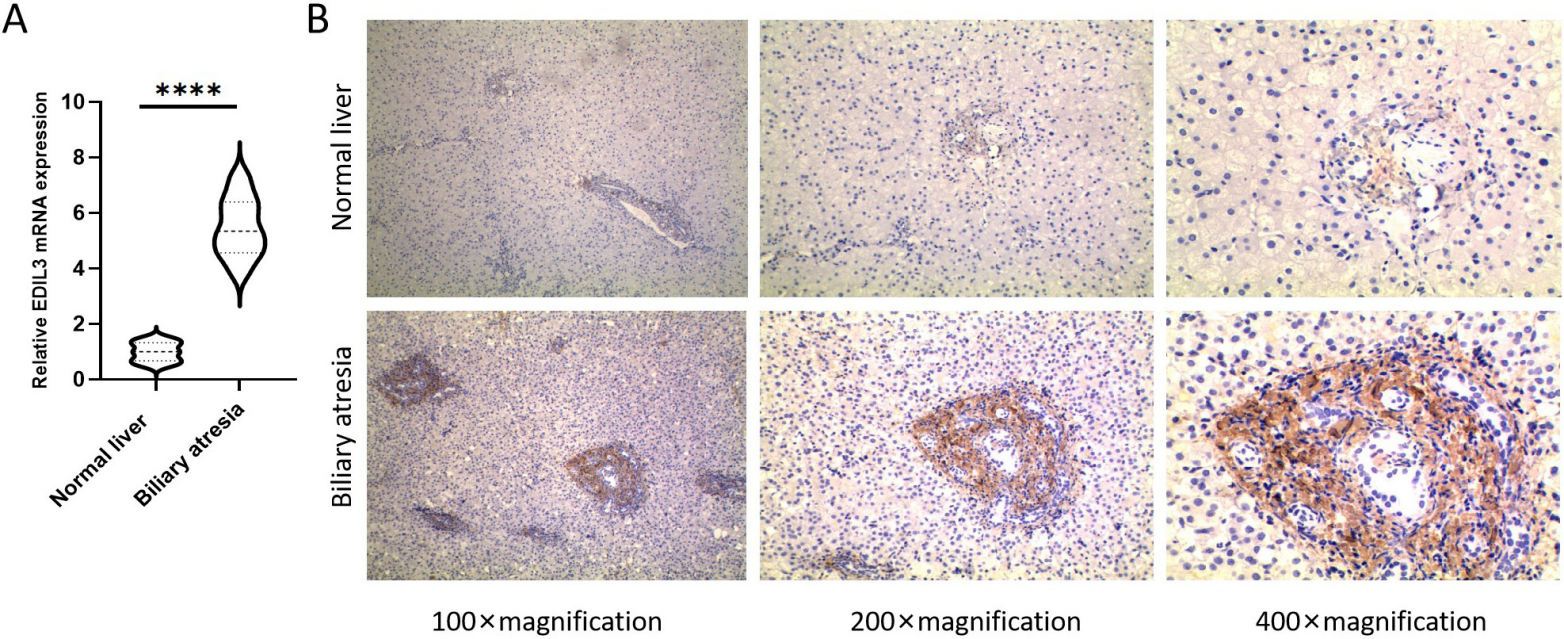
High expression of EDIL3 in BA liver tissues. **(A)** RT-qPCR results showing that EDIL3 mRNA levels in BA liver tissues were significantly greater than those in normal liver tissues. **(B)** IHC results indicated that the EDIL3 protein was expressed mainly in the cytoplasm and that its expression in BA liver tissues was significantly greater than that in normal liver tissues. ****P < 0.0001.

Real-time quantitative PCR analysis of 10 BA and 10 normal liver tissues revealed that the relative expression level of EDIL3 mRNA in BA liver tissue was significantly greater than that in the normal control group (Figure 5A). At the protein level, IHC analysis of liver tissues from 229 BA patients and 40 controls revealed that the EDIL3 protein was expressed primarily in the cytoplasm of hepatocytes, with no expression in the nucleus, and that its expression in BA liver tissue was significantly greater than that in normal liver tissue (Figure 5B). The IHC results revealed positive staining for EDIL3 in the cytoplasmic region, indicating its synthesis phase; after secretion, the mature protein localizes to the cell membrane and extracellular regions, which is consistent with the subcellular localization of EDIL3. This dual localization is typical of secretory proteins involved in signal transduction. In the BA group, the percentage of patients with high EDIL3 protein expression was 58.9%, whereas in the control group, it was only 12.5%, with a significant difference between the two groups (Table 1). Chi-square analysis revealed that the expression level of EDIL3 was significantly correlated with clinical indicators such as weight, bilirubin, alanine aminotransferase, albumin, lactate dehydrogenase, the international normalized ratio (INR), the neutrophil count, the native liver survival time, and the PELD score (Table 2).

**TABLE 1:** Comparison of EDIL3 protein expression between biliary atresia liver tissue and control tissue.

Groups	Total	EDIL3 high expression		EDIL3 low expression		*P*-value
		*n*	%	*n*	%	
Biliary atresia	229	135	58.9	94	41.1	<0.001
Normal liver	40	5	12.5	35	87.5	

**TABLE 2:** Relationships between EDIL3 expression and the clinical characteristics of BA patients.

Parameters	Native liver survival	Native liver death	Overall	*P*-value
	(*N* = 115)	(*N* = 114)	(*N* = 229)	
**Gender**				
Boy	56 (48.7%)	63 (55.3%)	119 (52.0%)	0.389
Girl	59 (51.3%)	51 (44.7%)	110 (48.0%)	
**Age_day**				
Mean (SD)	60.0 (15.8)	62.6 (16.0)	61.3 (15.9)	0.217
Median [Min, Max]	57.0 [36.0, 106]	60.5 [37.0, 108]	59.0 [36.0, 108]	
**Weight_kg**				
Mean (SD)	6.65 (1.12)	6.94 (1.02)	6.79 (1.08)	0.042**
Median [Min, Max]	6.90 [5.00, 8.40]	7.22 [5.00, 8.40]	7.00 [5.00, 8.40]	
**TB**				
Mean (SD)	156 (40.7)	207 (46.3)	182 (50.4)	<0.001**
Median [Min, Max]	157 [11.1, 250]	203 [124, 386]	180 [11.1, 386]	
**DB**				
Mean (SD)	109 (28.3)	129 (35.1)	119 (33.4)	<0.001**
Median [Min, Max]	108 [23.7, 195]	130 [9.40, 281]	121 [9.40, 281]	
**IB**				
Mean (SD)	49.8 (26.0)	67.9 (37.2)	58.8 (33.2)	<0.001**
Median [Min, Max]	45.0 [14.9, 198]	58.5 [25.6, 290]	51.9 [14.9, 290]	
**ALT**				
Mean (SD)	148 (101)	175 (128)	162 (116)	0.073
Median [Min, Max]	126 [7.00, 481]	137 [28.0, 632]	129 [7.00, 632]	
**AST**				
Mean (SD)	215 (126)	284 (194)	250 (166)	0.002**
Median [Min, Max]	195 [30.0, 929]	234 [69.0, 1120]	211 [30.0, 1120]	
**ALP**				
Mean (SD)	562 (161)	576 (197)	569 (179)	0.557
Median [Min, Max]	538 [198, 1300]	561 [142, 1190]	547 [142, 1300]	
**GGT**				
Mean (SD)	579 (482)	533 (422)	556 (453)	0.442
Median [Min, Max]	420 [96.0, 2560]	423 [57.0, 2040]	420 [57.0, 2560]	
**ALB**				
Mean (SD)	40.4 (18.4)	36.8 (3.88)	38.6 (13.4)	0.041**
Median [Min, Max]	39.0 [26.8, 233]	37.3 [20.0, 44.0]	38.0 [20.0, 233]	
**LDH**				
Mean (SD)	317 (174)	368 (174)	342 (176)	0.028**
Median [Min, Max]	310 [21.3, 1140]	341 [21.3, 1350]	326 [21.3, 1350]	
**INR**				
Mean (SD)	0.948 (0.0824)	1.10 (0.281)	1.03 (0.220)	<0.001**
Median [Min, Max]	0.960 [0.800, 1.08]	1.07 [0.880, 3.87]	1.02 [0.800, 3.87]	
**FIB**				
Mean (SD)	1.98 (0.698)	1.95 (0.566)	1.96 (0.634)	0.735
Median [Min, Max]	2.00 [0.710, 5.70]	1.96 [0.900, 3.51]	1.96 [0.710, 5.70]	
**WBC**				
Mean (SD)	6.78 (6.57)	5.78 (5.97)	6.28 (6.29)	0.228
Median [Min, Max]	2.45 [1.32, 25.2]	2.14 [1.45, 25.2]	2.25 [1.32, 25.2]	
**Neutrophil**				
Mean (SD)	6.16 (3.58)	7.56 (4.29)	6.86 (4.00)	0.008**
Median [Min, Max]	5.64 [0.990, 14.8]	6.74 [0.990, 19.4]	6.44 [0.990, 19.4]	
**Lymphocyte**				
Mean (SD)	5.97 (4.85)	5.92 (5.51)	5.95 (5.18)	0.933
Median [Min, Max]	4.36 [0.620, 19.6]	4.40 [0.420, 44.1]	4.36 [0.420, 44.1]	
**Monocyte**				
Mean (SD)	2.88 (2.60)	3.50 (3.21)	3.19 (2.93)	0.111
Median [Min, Max]	1.56 [0.430, 11.6]	1.93 [0.410, 14.2]	1.57 [0.410, 14.2]	
**PLT**				
Mean (SD)	415 (166)	404 (161)	410 (164)	0.603
Median [Min, Max]	408 [105, 967]	408 [105, 867]	408 [105, 967]	
**Cre**				
Mean (SD)	15.9 (3.05)	16.5 (4.02)	16.2 (3.56)	0.258
Median [Min, Max]	15.0 [9.00, 27.0]	15.0 [11.0, 33.0]	15.0 [9.00, 33.0]	
**Operative_time_min**				
Mean (SD)	198 (49.4)	191 (46.1)	194 (47.9)	0.265
Median [Min, Max]	188 [125, 450]	181 [121, 490]	187 [121, 490]	
**Length_of_day**				
Mean (SD)	16.1 (4.07)	16.4 (4.01)	16.2 (4.03)	0.598
Median [Min, Max]	15.0 [10.0, 38.0]	15.0 [10.0, 36.0]	15.0 [10.0, 38.0]	
**PELD**				
Mean (SD)	2.13 (2.03)	6.68 (2.81)	4.40 (3.34)	<0.001**
Median [Min, Max]	2.51 [−9.47, 4.33]	5.98 [4.41, 29.9]	4.33 [−9.47, 29.9]	
**Time_LT**				
Mean (SD)	28.1 (16.3)	20.2 (16.2)	24.2 (16.7)	<0.001**
Median [Min, Max]	26.0 [4.05, 60.4]	14.5 [2.80, 60.5]	21.0 [2.80, 60.5]	
**Surgery_method**				
Kasai	33 (28.7%)	45 (39.5%)	78 (34.1%)	0.114
Laparoscopy	82 (71.3%)	69 (60.5%)	151 (65.9%)	
**EDIL3**				
High	28 (24.3%)	107 (93.9%)	135 (59.0%)	<0.001**
Low	87 (75.7%)	7 (6.1%)	94 (41.0%)	

TB, total bilirubin; DB, direct bilirubin; IB, indirect bilirubin; ALT, alanine transaminase; AST, aspartate aminotransferase; ALP, alkaline phosphatase; GGT, c-Glutamyltransferase; ALB, albumin; LDH, lactate dehydrogenase; INR, international normalized ratio; FIB, fibrinogen; WBC, white blood cell count; PLT, platelet; Cre, serum creatinine; PELD, pediatric end-stage liver disease; Time_LT, autologous liver survival time.

**P < 0.01.

### 3.6 Correlation of EDIL3 expression with prognosis in children with BA

**FIGURE 6:**
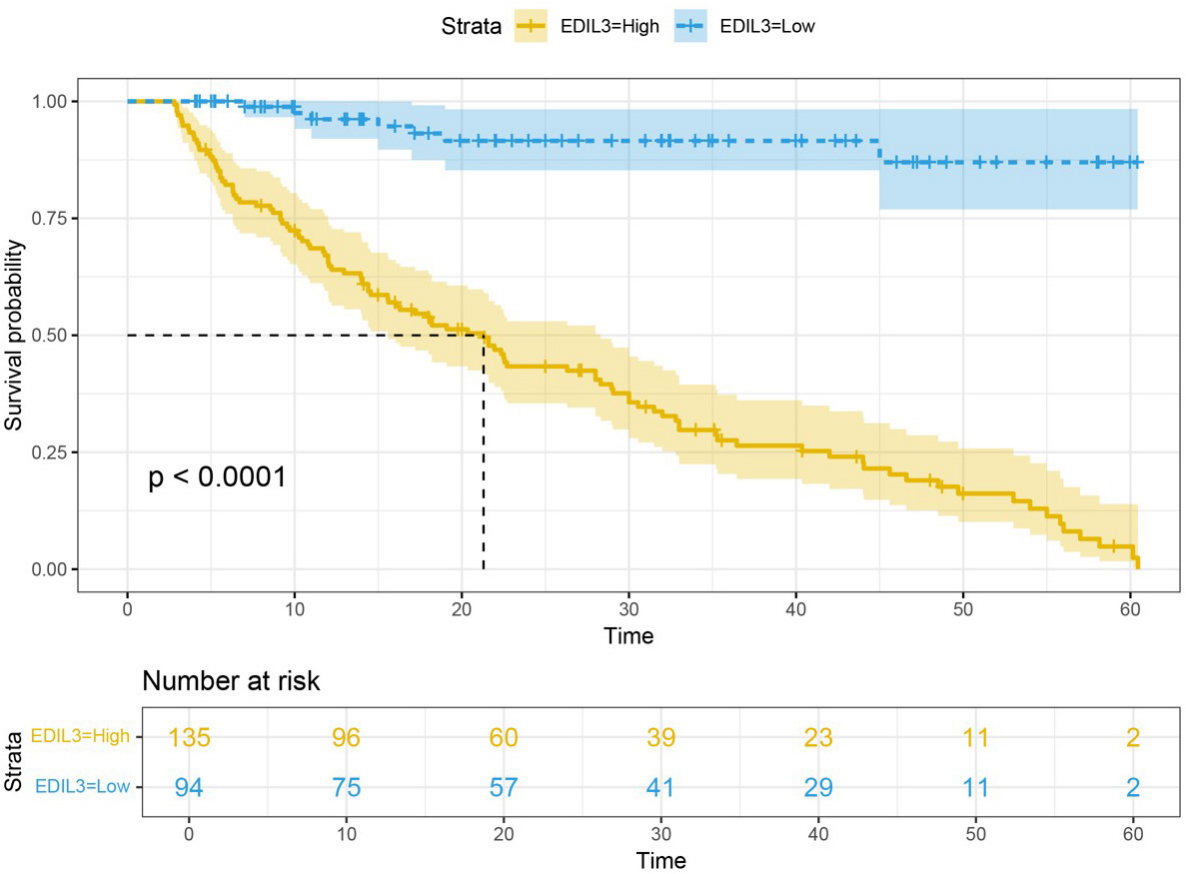
Kaplan-Meier survival curve of autologous liver survival in biliary atresia patients. Patients with high EDIL3 expression had a significantly lower overall survival rate than did those with low EDIL3 expression (*P* < 0.0001).

The 229 BA patients were divided into high EDIL3 expression and low EDIL3 expression groups on the basis of their IHC scores. Kaplan-Meier survival analysis revealed that the postoperative native liver survival rate of patients in the low-expression group was significantly greater than that of patients in the high-expression group; log-rank tests revealed that the survival curves of the two groups were significantly different (Figure 6). Univariate and multivariate Cox proportional hazards regression analyses revealed that high EDIL3 expression was an independent adverse prognostic factor affecting native liver survival in BA patients post-Kasai surgery (Table 3).

**TABLE 3:** Cox regression analysis of native liver survival.

Parameters	Univariable analysis			Multivariable analysis		
	Hazard ratio (95% CI)		*P*-value	Hazard ratio (95% CI)		*P*-value
Age	1.018	(1.004–1.032)	0.014	1.005	(0.994–1.016)	0.35
Weight	1.336	(1.059–1.686)	0.015	1.178	(0.973–1.425)	0.092
TB	1.005	(0.996–1.015)	0.252			
DB	0.997	(0.989–1.005)	0.405			
IB	0.995	(0.986–1.003)	0.245			
ALT	1	(0.997–1.002)	0.811			
AST	1.001	(0.999–1.003)	0.273			
ALP	1	(0.999–1.002)	0.8			
GGT	1	(0.999–1)	0.131			
ALB	0.981	(0.912–1.055)	0.6			
LDH	0.999	(0.998–1.001)	0.205			
INR	0.117	(0.011–1.213)	0.072			
FIB	0.704	(0.462–1.071)	0.101			
WBC	1.004	(0.963–1.047)	0.855			
Neutrophil	1.039	(0.944–1.144)	0.436			
Lymphocyte	1.025	(0.983–1.069)	0.244			
Monocyte	0.973	(0.855–1.107)	0.68			
PLT	1	(0.998–1.001)	0.762			
Creatinine	0.999	(0.944–1.058)	0.98			
PELD	1.328	(1.057–1.669)	0.015	1.086	(1.048–1.127)	0.01
EDIL3	5.794	(2.413–13.908)	0.001	9.379	(4.269–20.608)	0.001

TB, total bilirubin; DB, direct bilirubin; IB, indirect bilirubin; ALT, alanine transaminase; AST, aspartate aminotransferase; ALP, alkaline phosphatase; GGT, γ-Glutamyltransferase; ALB, albumin; LDH, lactate dehydrogenase; INR, international normalized ratio; FIB, fibrinogen; WBC, white blood cell count; PLT, platelet; PELD, pediatric end-stage liver disease.

## 4 Discussion

Through integrated bioinformatics analysis and clinical tissue validation, this study revealed many changes in gene expression in biliary atresia liver tissue, successfully identifying EDIL3 as a novel gene with a potentially key role in BA-related liver fibrosis. First, we identified 3706 DEGs in BA liver tissue, with a significantly greater number of upregulated genes than downregulated genes, suggesting widespread gene expression activation and reprogramming in the BA liver. Functional enrichment analysis further revealed that these genes were involved mainly in biological processes such as cell cycle regulation, DNA replication, transcription regulation, and extracellular matrix organization and were significantly enriched in KEGG pathways related to viral infection, the immune inflammatory response, the ECM-receptor interaction, and the PI3K-AKT signaling pathway. These findings match those of other studies, supporting the core roles of abnormal cell proliferation, immune dysregulation, and fibrotic responses in the pathogenesis of BA (13, 14, 19, 20). Second, through PPI network analysis and multialgorithm cross-screening, we ultimately identified 7 core genes, among which BRCA1, BRCA2, and TOP2A are closely related to DNA damage repair and cell cycle checkpoint regulation (21–23), suggesting that genomic instability and cell cycle dysregulation play important roles in the pathological process of BA. BUB1B is associated mainly with DNA damage repair and the progression of malignant tumors such as renal cancer, gastric cancer, and lung adenocarcinoma (24–27). HSP90AA1 plays crucial roles in the regulation of DNA damage, cell cycle control, gene expression, and carcinogenesis. Moreover, HSP90AA1 can promote cancer cell proliferation, metastasis, invasion, and epithelial-mesenchymal transition, suggesting that this protein may serve as a potential target for cancer therapy (28). PLK4 is essential for cell cycle progression and mitosis, and has been demonstrated to promote proliferation, invasion, and epithelial-mesenchymal transition (EMT) in various cancer types (29). The aforementioned genes may be involved in tissue fibrosis. However, EDIL3 was the most significantly upregulated gene in BA tissues, and how EDIL3 functions in the progression of liver fibrosis in BA patients remains unclear, making it the focus of this research.

Extracellular matrix proteins are major components of the extracellular microenvironment and play important roles in cell growth, invasion, migration, adhesion, and inflammatory responses (30). However, if tissues and organs are repeatedly damaged for various reasons, abnormal repair and pathological fibrosis can occur. Keeping this mechanism active might cause cholangiocytes to grow too much, ultimately impairing organ function and leading to systemic diseases. EDIL3 (also known as Del-1) is a secretory extracellular matrix protein that has been shown in previous studies to play important roles in cell adhesion, angiogenesis, and inflammation regulation (31–33). Additionally, EDIL3 promotes endocytosis and programmed cell death, regulating immune cell adhesion through its interaction with leukocyte-specific integrins (34–36). However, the potential role of EDIL3 in the progression of BA-related liver fibrosis remains unclear. This study first systematically revealed the high expression state of EDIL3 in the context of BA and found through GSEA that it was significantly positively correlated with the activation of apoptosis and the PI3K-AKT signaling pathway. We speculate that in the pathological microenvironment of BA, abnormally high expression of EDIL3 may activate the downstream PI3K-AKT signaling pathway by binding to cell surface integrin receptors (αvβ3), thereby regulating the balance of survival, proliferation, and apoptosis of cholangiocytes. Sustained activation of this mechanism may lead to abnormal proliferation of cholangiocytes and fibrotic responses, thereby promoting the progression of intrahepatic biliary obstruction and liver injury. This hypothesis is supported by recent studies showing that EDIL3 regulates hepatic stellate cell activation via the integrin αvβ3-ERK1/2-RUNX2 axis in metabolic dysfunction-associated steatotic liver disease (37), indicating that EDIL3-integrin interactions may be a common mechanism driving liver fibrosis in different liver diseases.

Interestingly, our bioinformatics analysis revealed that the subcellular localization of EDIL3 was primarily at the cell membrane and in the extracellular space, whereas the results of the immunohistochemical experiments revealed that EDIL3 was expressed in the cytoplasm. EDIL3 is a secreted glycoprotein that is synthesized in the endoplasmic reticulum (ER), processed in the Golgi apparatus, and ultimately transported to the ECM or presented on the cell membrane via integrin binding. During its biosynthesis, EDIL3 transiently localizes to the cytoplasm, particularly within the secretory compartments of the cell. IHC, which detects protein expression in fixed tissue sections, often captures proteins at various stages of their lifecycle, including during synthesis or intracellular storage. Therefore, the cytoplasmic staining observed via IHC likely reflects the presence of EDIL3 during its synthesis or prior to its secretion. Moreover, under pathological conditions such as BA, where there is significant endoplasmic reticulum stress, inflammation, and dysregulated extracellular matrix remodeling, the secretion process may be impaired or delayed. This could lead to the intracellular accumulation of EDIL3, contributing to the prominent cytoplasmic staining observed in hepatocytes and cholangiocytes. Thus, the IHC results do not contradict bioinformatics-based subcellular localization but rather complement it by illustrating the intracellular phase of EDIL3 in a disease context. This highlights the importance of considering both the canonical localization and the pathological context when interpreting protein expression patterns.

Research by Sun et al. (38) revealed that EDIL3 is highly expressed in hepatocellular carcinoma and is associated with poor patient prognosis, suggesting that it could serve as a novel prognostic marker and therapeutic target for hepatocellular carcinoma. Wei et al. recently discovered that EDIL3 regulates HSC activation by binding to integrin αvβ3, affecting fibrosis in metabolic dysfunction-associated steatotic liver disease (MASH), thus providing a potential therapeutic avenue for MASH and liver fibrosis (37). In our previous study, we employed WGCNA to identify LECT2 as a gene closely associated with liver fibrosis in BA, with its expression significantly upregulated in BA liver tissues. Elevated LECT2 expression indicates a poor prognosis in BA patients and serves as an independent prognostic factor affecting native liver survival. Thus, LECT2 may function as a valuable prognostic biomarker for children with BA. Detection of LECT2 expression in the liver tissues of BA patients could assist in determining the optimal timing for liver transplantation and evaluating clinical outcomes (12). These findings align with our findings; in clinical samples of BA, we observed high expression of EDIL3 in BA liver tissue through qPCR and IHC methods and reported that its high expression was significantly negatively correlated with the survival rate of patients’ native livers. Multivariate Cox regression analysis further confirmed that EDIL3 is an independent risk factor affecting the prognosis of BA patients. These results not only highlight the importance of EDIL3 in BA but also suggest its potential as a prognostic marker or therapeutic target. On the basis of the above findings, we observed that both LECT2 and EDIL3 are upregulated in BA and are associated with poorer native liver survival; however, yet they likely function through distinct mechanisms: LECT2 may play a key role via immune and metabolic regulation, whereas EDIL3 appears to exert significant effects through integrin-PI3K-Akt signaling and ECM remodeling. These results reinforce the novelty of EDIL3 as an independent prognostic and mechanistic factor. Notably, the combination of EDIL3 and LECT2 may further improve the prognostic prediction accuracy for BA patients, which warrants further validation in large-scale multicenter studies.

Although this study achieved meaningful results in bioinformatics mining and preliminary clinical validation, certain limitations should be acknowledged. This study did not fully account for the influence of clinical heterogeneity, such as stratification by age of surgical children within 180 days (<90 days vs. 90–180 days), the presence of postoperative infections, and compliance with follow-up, which may affect the association between EDIL3 levels and prognosis. Further stratified analyses are needed required for validation. Additionally, the potential batch processing of public database data is a factor influencing the results. More importantly, functional validation of the specific molecular mechanisms by which EDIL3 regulates the PI3K-AKT pathway is lacking. Future work should include EDIL3 knockout or overexpression in BA-relevant cell models to examine its direct effects on AKT phosphorylation, cell proliferation, and apoptosis. While the current study revealed an association between EDIL3 and PI3K-AKT signaling in BA-related liver fibrosis, its primary value lies in establishing a theoretical foundation and providing experimental support for subsequent in-depth mechanistic exploration. Additionally, exploring the potential of EDIL3 as a therapeutic target (using neutralizing antibodies or small-molecule inhibitors to block the EDIL3-integrin αvβ3 interaction) may provide new strategies for the treatment of BA-related liver fibrosis. While our integrated analysis strongly suggests a role for EDIL3 in BA-related fibrosis, we note that the connection in this study remains indirect. We did not directly correlate EDIL3 expression levels with established histological or molecular (α-SMA, collagen I) markers of fibrosis within the same patient cohort. The profibrotic role of EDIL3 is inferred from its association with the PI3K-AKT pathway, its prognostic value for liver survival, and supportive evidence from other liver fibrosis models. Future studies are essential to directly demonstrate this link. This should include costaining of EDIL3 with α-SMA in BA liver sections to assess spatial correlation with activated HSCs, as well as *in vitro* experiments using cholangiocyte or stellate cell models to test whether EDIL3 manipulation directly affects collagen deposition or other fibrosis-related endpoints.

## 5 Conclusion

This study systematically analyzed the transcriptomic landscape of BA liver tissues and identified EDIL3 as a new key gene implicated in liver fibrosis. EDIL3 likely contributes to fibrogenesis by influencing cholangiocyte fate via the PI3K-AKT signaling pathway. These findings increase our understanding of the molecular mechanisms of BA and establish a foundation for the development of EDIL3-based diagnostic or therapeutic strategies.

## Data Availability

The datasets presented in this study can be found in online repositories. The names of the repository/repositories and accession number(s) can be found in the article/Supplementary material.

## Footnotes

1
https://www.ncbi.nlm.nih.gov/geo/

2
https://davidbioinformatics.nih.gov/

3
https://string-db.org/
